# Unique T Cells with Unconventional Cytokine Profiles Induced in the Livers of Mice during *Schistosoma mansoni* Infection

**DOI:** 10.1371/journal.pone.0082698

**Published:** 2013-12-16

**Authors:** Keishi Adachi, Yoshio Osada, Risa Nakamura, Koji Tamada, Shinjiro Hamano

**Affiliations:** 1 Department of Parasitology, Institute of Tropical Medicine (NEKKEN), Nagasaki University, Nagasaki, Japan; 2 Global Center of Excellence Program, Nagasaki University, Nagasaki, Japan; 3 Department of Immunology and Parasitology, The University of Occupational and Environmental Health, Yahatanishi-ku, Kitakyushu, Japan; 4 Department of Immunology and Cell Signaling Analysis, Yamaguchi University Graduate School of Medicine, Ube, Japan; Federal University of São Paulo, Brazil

## Abstract

During infection with *Schistosoma*, serious hepatic disorders are induced in the host. The liver possesses unique immune systems composed of specialized cells that differ from those of other immune competent organs or tissues. Host immune responses change dramatically during *Schistosoma mansoni* infection; in the early phase, Th1-related responses are induced, whereas during the late phase Th2 reactions dominate. Here, we describe unique T cell populations induced in the liver of mice during the period between Th1- and Th2-phases, which we term the transition phase. During this phase, varieties of immune cells including T lymphocytes increase in the liver. Subsets of CD4^+^ T cells exhibit unique cytokine production profiles, simultaneously producing both IFN-γ and IL-13 or both IFN-γ and IL-4. Furthermore, cells triply positive for IFN-γ, IL-13 and IL-4 also expand in the *S. mansoni*-infected liver. The induction of these unique cell populations does not occur in the spleen, indicating it is a phenomenon specific to the liver. In single hepatic CD4^+^ T cells showing the unique cytokine profiles, both T-bet and GATA-3 are expressed. Thus, our studies show that *S. mansoni* infection triggers the induction of hepatic T cell subsets with unique cytokine profiles.

## Introduction

The liver is characterized by a unique micro-anatomical and immunological environment [1]–[3]. It contains Kupffer cells and a large number of resident lymphocytes, including NK cells and NKT cells, whose immunological environment unlike that of any other organs or tissues [2], [4]. Although a large amount of enteric and systemic blood-borne antigens constitutively enter into, are trapped and accumulated in the liver, immune responses are tightly regulated in a homeostatic state, and many hepatic lymphocytes show ‘activated-yet-resting’ phenotypes. Important pathogens, for example, the hepatitis C virus and malaria parasites, take advantage of the liver's immune condition, circumvent immunity, and establish chronic infections [5], [6]. In contrast, some microorganisms such as the hepatitis B virus induce severe immune reactions in a liver, resulting in fulminant hepatitis [6], [7]. Why liver-specific immune competent cells show such uncommon and inconsistent features remains unresolved.

Parasitic worms are important pathogens, affecting the health of roughly 2 billion people living mostly in tropical and subtropical environments [8]. One specific genus within Platyhelminths, the *Schistosoma*, constitutes a major health burden for human populations in many parts of the world. In 2009, 239 million people were infected with schistosomes, 85% of them in sub-Saharan Africa, where approximately 150,000 deaths per year were attributable to the worms [9]–[11].

Following skin penetration by cercariae, schistosomes migrate via the blood to the hepatic portal vein, where they rapidly mature and mate. Egg production begins 4–6 weeks following infection. A single female parasite is estimated to produce 300 eggs per day, many of which enter the liver via the blood stream. The liver, therefore, is a primary organ of pathogenic injury and subsequent granulomatous tissue damage, and pathogenesis in liver is the most important for etiology, although chronic inflammation is induced in several other organs [12]–[14].

Following infection with *Schistosoma mansoni* (*S. mansoni*) cercariae, the host immune responses progress through at least two phases. Th1-related responses are induced in the early phase (3–5 weeks postinfection, PI). As the parasites begin oviposition (4.5–6 weeks PI), the Th1 components are gradually down-regulated, and strong Th2 reactions are induced [12]. Intensive studies of the Th2 phase have been conducted, as it is during this period that hepatic pathology becomes prominent. However, the roles of immune reactions during early Th1 phase, especially in the liver, have been little investigated [12], [14]–[17]. It has been previously reported that the balance between Th1 and Th2 responses are important for the severity of schistosomiasis, and that ‘smooth’ phase transitions are observed in hosts not showing serious symptoms [12], [13], [18]–[20].

It is conventionally believed that Th1 and Th2 reciprocally inhibit their generations, and that one helper T cell does not normally produce both Th1- and Th2-related cytokines, particularly IFN-γ and IL-4, simultaneously, [21], [22]. Therefore, we hypothesized that unknown cellular and/or molecular mechanisms ‘bridging’ Th1 and Th2 generation occur in the liver between Th1 and Th2 phase (‘transition’ phase) of *S. mansoni* infection. In order to test this hypothesis, we analyzed the immune responses induced in the liver following *S. mansoni* infection, using mouse cercarial infection models.

Here we show that unique CD4^+^ T cell populations that simultaneously produce Th1- and Th2-cytokines, combinations of “IFN-γ and IL-13” and “IFN-γ and IL-4”, accumulate in the liver, but not in the spleen, during the transition phase of *S. mansoni* infection. Moreover, some of these unique populations acquire the potential for secreting the three cytokines concomitantly. Our present observations provide new insights into the mechanisms underlying the pathogenesis of schistosomiasis. Furthermore, these findings point to a new concept in T cell biology; the antagonism between Th1 and Th2 responses can be resolved in some immunological conditions.

## Materials and Methods

### Mice

Female BALB/c mice (6–10 week-old) and C57BL/6 mice (6–10 week-old) were purchased from SLC (Shizuoka, Japan), and maintained under specific pathogen-free conditions. Experiments were conducted with BALB/c mice unless otherwise specified.

### Maintenance of the parasite life cycle and infection of mice with *S. mansoni*

*S. mansoni* was maintained as previously described [23], [24]. Mice were anesthetized and percutaneously infected with 25 *S. mansoni* cercariae as previously described [25]. Egg burden was microscopically observed in feces and the caudate lobe of the liver, and in most cases, began at 4–5 weeks PI (data not shown), as previously reported [12].

### Intracellular cytokine staining (ICS)

ICS technology was used to monitor cytokine production [26]. In brief, hepatic lymphocytes and splenocytes were prepared from mice at indicated weeks after the infection as previously described [27]–[29]. In each group, hepatic lymphocytes isolated from 3 mice were pooled in order to obtain sufficient cell numbers. These were then stimulated with immobilized anti-mouse CD3 (17A2, BioLegend) and anti-CD28 (E18, BioLegend) for 5 hours in the presence of brefeldin A. Cell surface molecules were stained with PE-Cy5-, PE-Cy7-, or Allophycocyanin (APC)-Cy7-conjugated anti-CD4 (GK1.5, BioLegend), APC-conjugated anti-CD8α (53-6.7, BioLegend), APC-conjugated pan-NK cell (DX5, BioLegend), PE-Cy7-conjugated anti-CD62L (MEL-14, BioLegend), PerCP-Cy5.5-conjugated anti-CD44 (IM7, BioLegend), PerCP-Cy5.5-conjugated anti-CD27 (LG.3A10, BioLegend), PerCP-Cy5.5-conjugated anti-CD197 (CCR7, 4B12, BioLegend), PE-Cy7-conjugated anti-CXCR5 (2G8, BD Biosciences), or PerCP-Cy5.5-conjugated anti-CD278 (ICOS, C398.4A, BioLegend). Fixation and permeabilization of the cells were conducted with 2% formaldehyde and 0.5% saponin, respectively. For the detection of intracellular cytokines, FITC-, PE-, or APC-conjugated, corresponding monoclonal antibodies were used (IL-4; 11B11, IFN-γ; XMG1.2, IL-5; TRFK5, BioLegend; IL-13; eBio13A, eBioscience). Flowcytometric analysis was conducted with FACSCalibur, FACSCanto II, or FACSVerse (BD Biosciences), and the data were analyzed with CellQuest (BD Biosciences) or FlowJo software (Tree Star, Inc.)**.** Culture medium was RPMI-1640 supplemented with 10 % FCS, 100 U/ml penicillin, 100 mg/ml streptomycin, 50 mM of 2-mercaptoethanol and 2 mM L-glutamine.

### Flowcytometric analysis of transcription factors

Flowcytometry was used for the analysis of transcription factors. Briefly, cell surface molecules were stained with fluorochrome-conjugated monoclonal antibodies as mentioned above. Fixation, permeabilization, and staining of the target transcription factors were conducted with FoxP3/Transcription Factor Staining Buffer Set (eBioscience) according to the manufacturer’s instructions. For the detection of the transcription factors, PerCP-Cy5.5-conjugated anti-T-box expressed in T-cells (T-bet, 4B10, BioLegend), PE-Cy7-conjugated anti-Gata-binding protein 3 (GATA-3, L50-823, BD Biosciences), or Alexa Fluor® 647 anti-Bcl-6 were used.

### Statistics

All data are shown as the mean values of more than three independent experiments. Significance between the control group and treated group was determined with Student’s unpaired *t*-tests. *P* values less than 0.05 were considered significant.

### Ethics Statement

All mouse experiments were conducted according to relevant national and international guidelines, and were approved by the Institutional Animal Care and Use Committee at Nagasaki University.

## Results

### Explosive increase of immune competent cells in the livers of *S. mansoni*-infected mice during the transition phase

**Figure 1:**
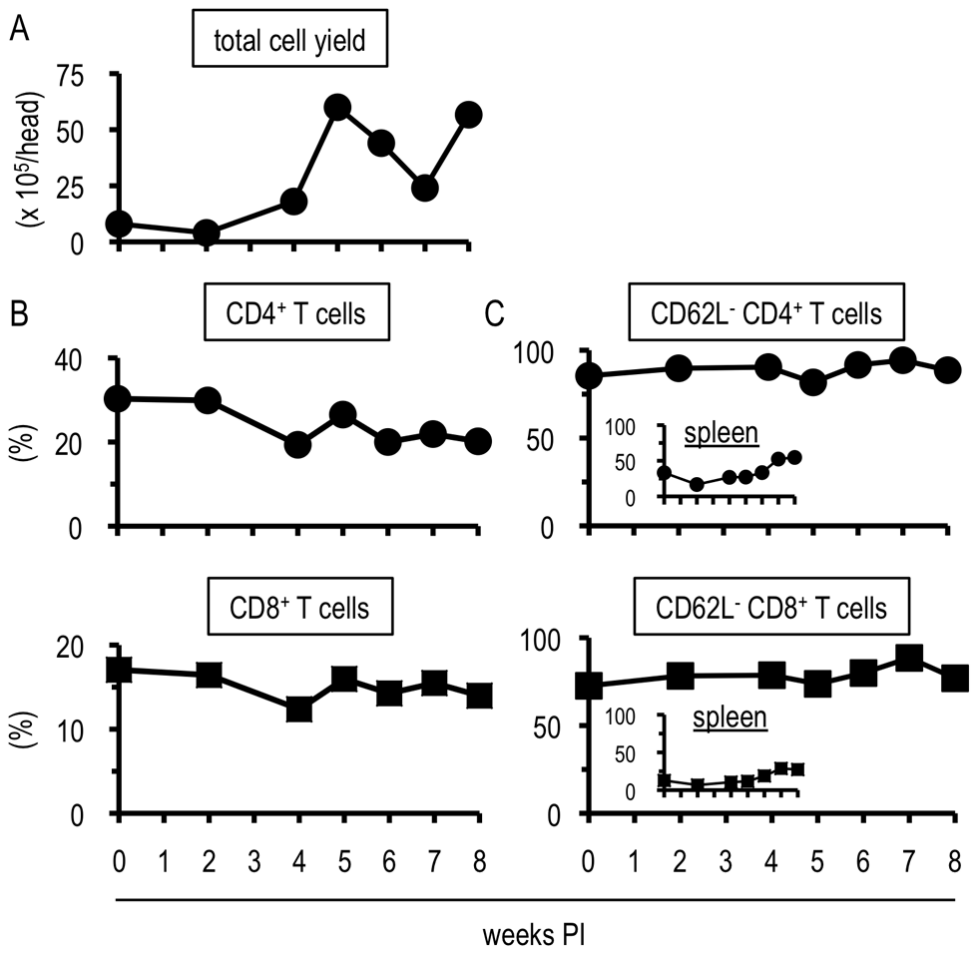
*Schistosoma mansoni* infection induced robust increase of immune competent cells in the liver. (A) Hepatic cells isolated from 3 mice were pooled and the cell number was calculated. (B and C) Flowcytometric analysis was conducted with the liver lymphocytes prepared in (A) or the splenocytes. The percentages in (B) represent the proportions in CD3-positive population, and those in (C) express the proportions in population of (B). (A-C) each shows one representative result of three independent experiments.

It has been reported that the balance between Th1- and Th2-related immune responses in a host infected with *S. mansoni* is closely related to the severity of the disease [12], [13], [18]–[20]. The most serious form of intestinal schistosomiasis is hepatic disorders [12]–[14]. This prompted us to analyze the immunological events induced in the liver during *S. mansoni* infection. First, we investigated the kinetics of the number of hepatic cells recruited into and/or expanded in the liver following cercarial infection. Hepatic cells were isolated at 0, 2, 4, 5, 6, 7, and 8 weeks PI. As shown in *Figure 1A*, cell yields began to increase at 4 weeks PI and peaked at 5–7 weeks PI, suggesting that significant numbers of cells infiltrated into and/or expanded in the liver during the transition phase of *S. mansoni* infection.

Next, we investigated the components of the cell populations that increased in the liver during the transition phase by flowcytometry. Consistent with previous reports [12], [14], [30], CD4^+^ T cells, CD8^+^ T cells, B cells, NK cells, and eosinophils were found (Fig. 1B, S1). Notably, most (≥ 70%) of CD4^+^ and CD8^+^ T cells exhibited an activated phenotype (CD62L-negative) in the liver, but not in the spleen, irrespective of the infection (Fig. 1C). Since total cell yield was dramatically increased after the infection (Fig. 1A), the absolute number of the activated T cells also increased (data not shown). Taken together, *S. mansoni* infection induced a marked increase in cell number including activated T cells in the liver particularly during the transition phase.

### Hepatic CD4^+^ T cells induced during the transition phase of *S. mansoni* infection demonstrate a unique potential for cytokine production

As cytokine environments, especially those of CD4^+^ helper T cells, play important roles in the generation of hepatic granulomatous lesions in intestinal schistosomiasis [12]–[14], [31]–[33], the cytokine profiles of the hepatic CD4^+^ T cells were analyzed. For this purpose, we conducted ICS experiments upon TCR-ligated cells, and found that *S. mansoni* infection elicited hepatic CD4^+^ T cells to produce TNF-α, IL-5, or IL-10. In contrast, little IL-17 production was observed (Fig. S2). The production of all cytokines investigated started after 4 weeks PI, the early transition phase. It is noteworthy that the kinetics of the T cell population positive for each cytokine showed individually distinct time-courses (Fig. S2). This suggests that *S. mansoni* infection confers a wide variety of cytokine production to hepatic CD4^+^ T cells, and that the local immune environment in the liver is closely related to fluke growth.

**Figure 2:**
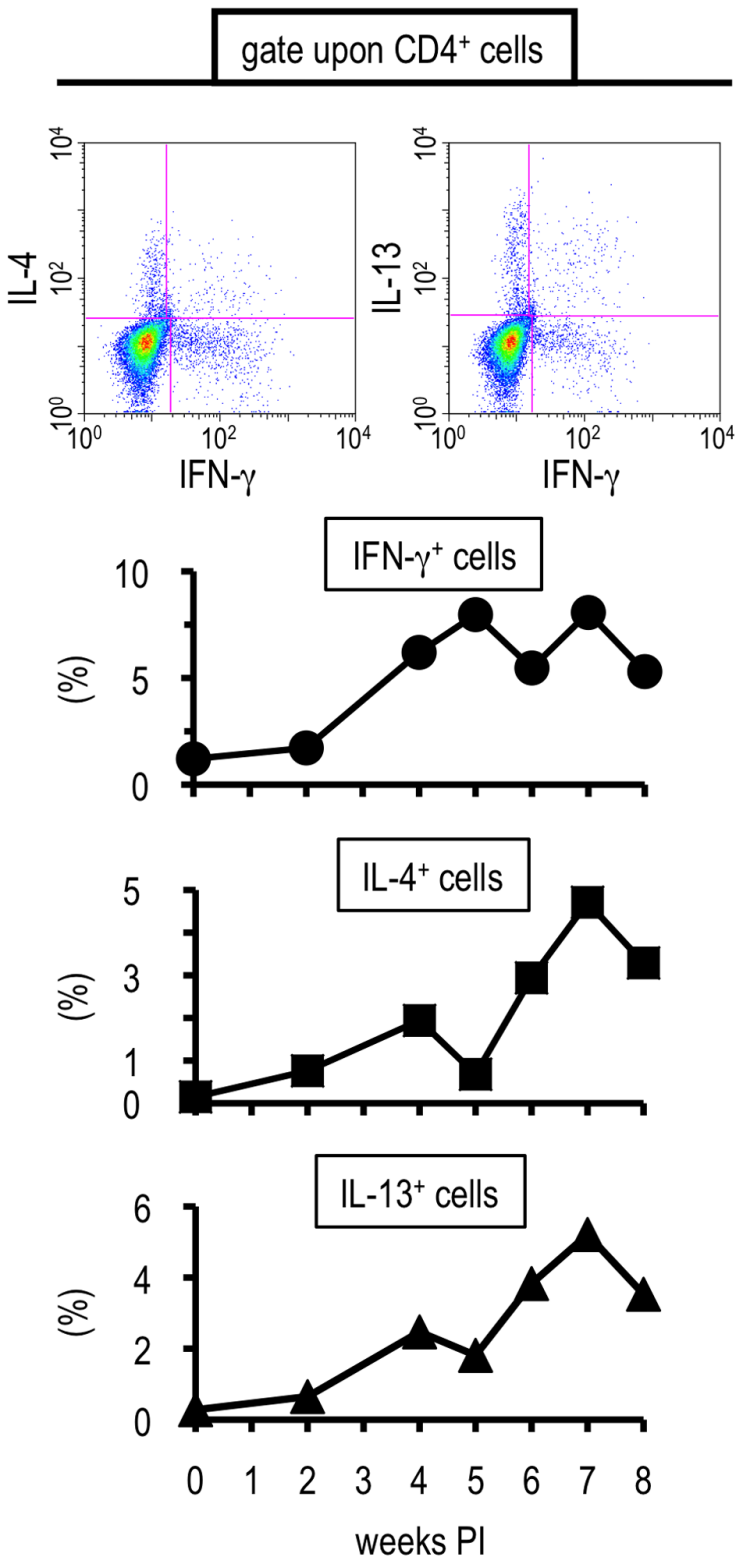
Th1 cells were induced in early and Th2 cells in late phase in the liver. Hepatic lymphocytes were isolated from *S. mansoni*-infected BALB/c mice at indicated time points, and their potential for producing IFN-γ, IL-4, or IL-13 was analyzed by ICS upon TCR ligation. Insets at the top represent one example using liver lymphocytes prepared at 4 weeks PI are shown. The percentages represent the proportions in CD4-positive population. Similar results were obtained in three independent experiments.

Next, we focused upon the typical Th1- and Th2-related cytokines, IFN-γ, IL-4, and IL-13. Similar to reports concerning systemic T cell reponses [12], [13], the increase of IFN-γ-producing CD4^+^ T cells began earlier than that of IL-4- or IL-13-producing cells in the liver (Fig. 2). Yet, unlike the systemic immune reactions previously reported [12], [13], CD4^+^ T cells secreting IFN-γ were not clearly down-regulated in the phase when Th2 cytokine-secreting T cells were increasing (Fig. 2).

**Figure 3:**
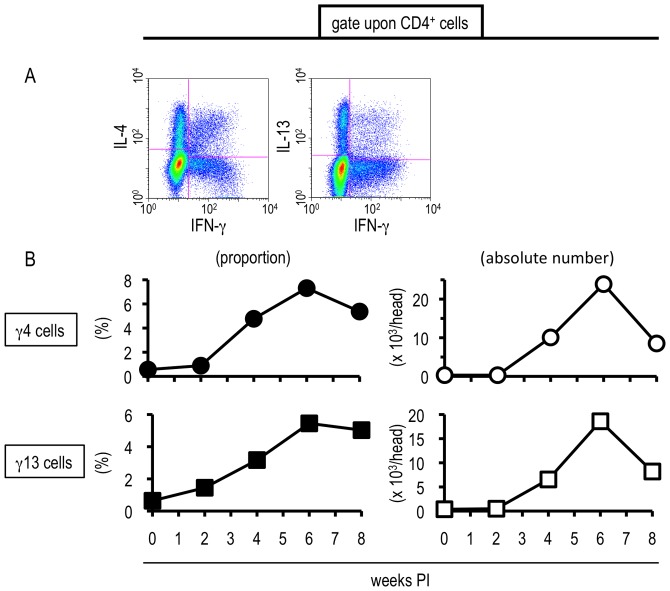
*S. mansoni* infection-induced hepatic T cells exhbited the potential to produce uncommon combinations of cytokines. Hepatic lymphocytes were isolated from *S. mansoni*-infected mice at indicated time points, and the proportions and absolute numbers of γ4 and γ13 cells were investigated by ICS. (A) One example using hepatic lymphocytes prepared at 6 weeks PI is displayed. (B) The percentages represent the proportions in CD4-positive population. This experiment is representative of three independent experiments.

Intriguingly, some hepatic CD4^+^ T cells isolated from *S. mansoni*-infected mice exhibited unique cytokine profiles, producing both IFN-γ and IL-4 (“γ4 cells”) or IFN-γ and IL-13 (“γ13 cells”) (Fig. 3A). Both populations began to increase their proportion and absolute numbers during the transition phase of the infection (Fig. 3B). Then, in the late phase, when strong Th2 responses were induced systemically, both γ4 and γ13 cells showed a tendency to decrease (Fig. 3B). Hepatic CD8^+^ T cells produced neither IL-4 nor IL-13 throughout the infection although obvious IFN-γ production was observed (Fig. S3, data not shown). Hence, populations exhibiting unique cytokine profiles were not observed within the CD8^+^ T cell population. This suggests that hepatic CD4^+^ T cells, but not CD8^+^ T cells, acquired unconventional cytokine productivities during *S. mansoni* infection, especially during the transition phase. In the spleen, neither γ4 nor γ13 cells were induced throughout the infection (Fig. S4), indicating that the induction of these unusual cell populations was not ubiquitously observed in the body, but were liver-specific immunological events. As shown in *Figure S5*, both γ4 and γ13 cells were induced not only in the livers of BALB/c but also in those of C57BL/6 mice during the transition phase of the infection. Therefore, the acquisition of the unique cytokine profiles was not restricted to a particular strain of congenic mice.

**Figure 4:**
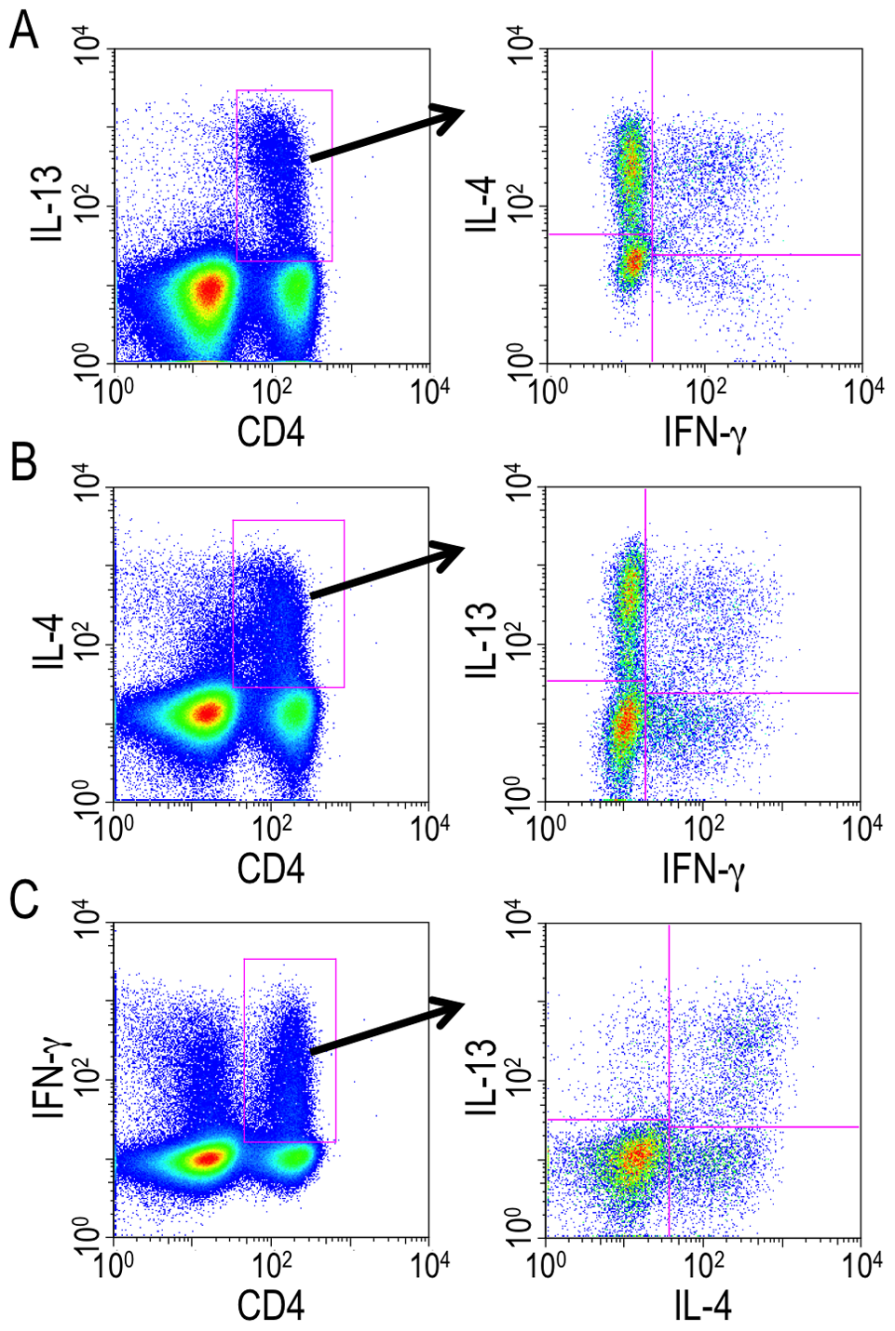
The unique hepatic T cells exhbited the ability to produce IFN-γ, IL-13, and IL-4 simultaneouly. Hepatic lymphocytes were isolated from *S. mansoni*-infected mice at 6 weeks PI and ICS was conducted after TCR stimulation. γ4 cells within IL-13-producing CD4^+^ T cell population (A), γ13 cells within IL-4-producing CD4^+^ T cell population (B), and IL-4- and IL-13-secreting cells within IFN-γ-producing CD4^+^ T cell population (C) were analyzed. Data shown are a representative of five independent experiments.

We next analyzed whether the unique hepatic T cell populations had the ability to produce IFN-γ, IL-4, and IL-13 simultaneouly. As shown in *Figure 4A*, γ4 cells were observed within the IL-13-producing CD4^+^ T cell population. γ13 cells were also detected within the IL-4-secreting cells (Fig. 4B). And within the IFN-γ^+^ population, CD4^+^ cells doubly positive for IL-4 and IL-13 were also found (Fig. 4C). Taken together, some proportion of the hepatic CD4^+^ T cells in the liver during the transition phase of the infection indeed acquired the capacity for producing IFN-γ, IL-13, and IL-4 simultaneously (“triple positive cells”).

In the absence of TCR stimulation ex vivo, the freshly isolated hepatic lymphocytes produced little cytokine (Fig. S6). Moreover, incubation of the hepatic lymphocytes on the plates coated with isotype matched control antibodies, instead of anti-CD3 and anti-CD28, rarely induced cytokine secretion (data not shown).

All of the results, collectively, suggested that during the transition phase of *S. mansoni* infection, hepatic CD4^+^ T cells acquire the potential to produce very unique combinations of cytokines; ‘co-prime’ Th1- and Th2-related cytokines.

### The unique hepatic CD4^+^ T cells demonstrate a production selectivity of Th2-related cytokines

**Figure 5:**
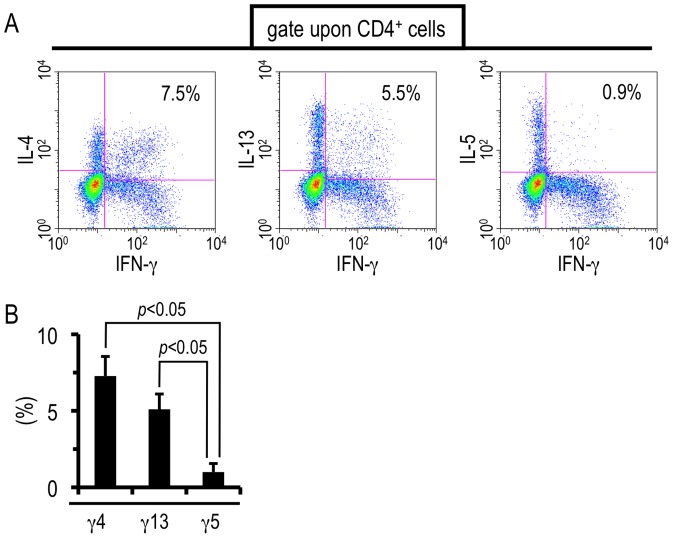
CD4^+^ T cells doubly producing IFN-γ and IL-5 were rarely induced after *S. mansoni* infection. Hepatic lymphocytes were isolated from *S. mansoni*-infected mice at 6 weeks PI and ICS was conducted upon TCR ligation. (A) One representative result is shown. The numbers in the insets represent the percentages of γ4, γ13, or γ5 in CD4-positive population. (B) Data represent the mean values + SD of three independent experiments.

Next, we investigated whether the unique liver T cells have the potential to produce Th2 cytokines other than IL-4 and IL-13. For this purpose, we selected IL-5 as a Th2-related cytokine other than IL-4 and IL-13, because infiltration of eosinophils into the liver and up-regulation of IL-5-producing CD4^+^ T cells were induced after *S. mansoni* infection, particularly during the transition phase (Fig. S1 and S2). TCR ligation elicited the simultaneous production of IFN-γ and IL-4 or IFN-γ and IL-13 from the hepatic CD4^+^ T cells isolated during the transition phase (Fig. 5A, Fig. 3). However, few cells produced IFN-γ and IL-5 concurrently although CD4^+^ T cells that solely secrete either IFN-γ or IL-5 were unambigously induced in the liver (Fig. 5B). Taken together, *S. mansoni* infection confers selectivity for producing Th2-related cytokines as well as the unique cytokine profiles mentioned above on hepatic CD4^+^ T cells.

### αβ T cells, but not γδ T cells, exhibit uncommon cytokine profiles upon *S. mansoni* infection

**Figure 6:**
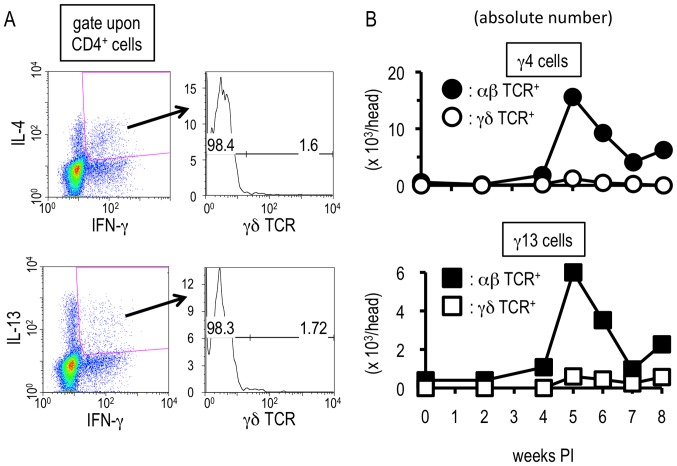
Hepatic αβ T cells are the responsible cells showing the unconventional cytokine profiles. Hepatic lymphocytes were isolated from *S. mansoni*-infected mice and flowcytometry was conducted after TCR ligation. (A) Expression levels of γδ TCR on CD4^+^ γ4 cells or γ13 cells were analyzed with the hepatic lymphocytes isoleted at 6 weeks PI. The values in the right insets indicate percentages of γδ TCR-positive or –negative population in CD4^+^ γ4 or γ13 cells. This experiment is a representative of four independent experiments. (B) The absolute numbers of γ4 cells (upper graph) or γ13 cells (lower graph) in αβ TCR- or γδ TCR-potitive population were investigated. Similar results were obtained in three independent experiments.

We analyzed the precise characters of the hepatic CD4^+^ T cell populations showing the unique cytokine secretion patterns. γδ T cells can act as producers of Th2- as well as Th1-related cytokines in some situations [34]–[37], and the liver is rich in γδ T cells [34]–[36], [38]. Therefore, we first analyzed whether γδ T cells could be γ13 and γ4 cells. As shown in *Figure 6A*, the unique T cell populations little expressed γδ TCR. Moreover, neither γ13 nor γ4 cells were observed within γδ TCR-positive population throughout the infection (Fig. 6B). In contrast, after *S. mansoni* infection, the αβ TCR-expressing population displayed the unique cytokine production patterns during the transition phase (Fig. 6B). Taken together, this suggests that *S. mansoni* infection confers uncommon capacities for cytokine production upon αβ T cells but not on γδ T cells during the transition phase.

Then, we characterized the hepatic γ4 and γ13 cells more precisely with several cell surface molecules. As shown in *Figure S7*, the majority of the both cell types exhibited CD62L-negative, CD44-positive, CD27-negative, and CCR7-negative phenotypes. This indicates that most of the hepatic γ4 and γ13 cells possesses the features resembling to effector memory T cells.

### DX5-negative as well as –positive cells displayed unique cytokine production patterns

**Figure 7:**
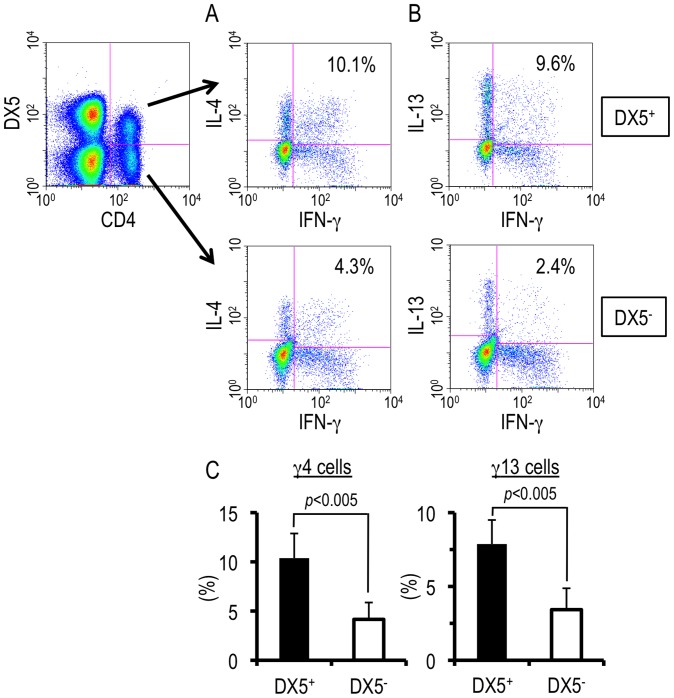
DX5-negative as well as –positive cells displayed the unique cytokine production patterns. (A and B) Hepatic lymphocytes were isolated from *S. mansoni*-infected mice and flowcytometric analysis was conducted upon TCR ligation at 6 weeks PI. The numbers in the insets represent percentages of γ4 (A) or γ13 (B) cell population in CD4^+^ DX5-positive or -negative population. This experiment is representative of five independent experiments. (C) Data represent the mean + SD of five independent experiments.

DX5, also known as integrin α2, is a mouse pan-NK cell marker. DX5-expressing T cells including classical *i*NKT cells, whose generation is restricted to CD1d and whose TCR is invariant, are abundant in the liver even in the homeostatic state [39]–[41] and rapidly increase in number in some circumstances, such as during *Plasmodium* spp. infection [27], [42]. It is well-known that both NKT cells and activated T cells have a high-potential for producing cytokines. This prompted us to analyze whether DX5 is expressed upon hepatic γ4 and γ13 cells induced during the transition phase of *S. mansoni* infection, i.e., whether the γ4 and γ13 cells consist of a single population or not. As shown in *Figure 7A*, γ4 cells were observed within both DX5-negative and -positive cell populations. This is also the case for γ13 cells (Fig. 7B). The ratio of γ4 or γ13 cells within DX5-positive population was higher than that within DX5-negative population (Fig. 7C). These results suggest that the unique hepatic CD4^+^ T cell populations could be divided into two sub-populations; DX5-negative ‘conventional’ T cells and DX5-positive T cells, including NKT cells.

### T-bet and GATA-3 were co-expressed within a single γ4 or γ13 cell

**Figure 8:**
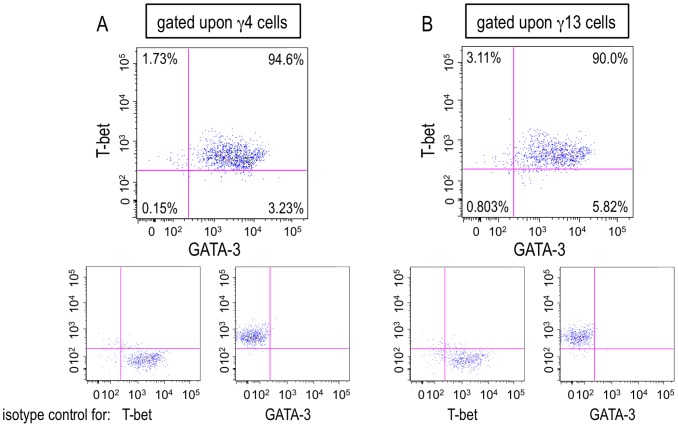
Both T-bet and GATA-3 were expressed in a single hepatic γ4 or γ13 cell. (A and B) Hepatic lymphocytes were isolated from *S. mansoni*-infected mice and the expressions of T-bet and GAT-3 were analyzed by flowcytometry after TCR stimulation at 6 weeks PI. The numbers in the upper, large insets represent percentages of each population divided by the expressions of T-bet and GATA-3 in γ4 (A) or γ13 (B) cells. The lower, small insets represent the data using isotype control antibodies. Similar results were obtained in three independent experiments.

T-bet and GATA-3 are the crucial transcription factors for Th1 and Th2, respectively. It has been believed that they counteract reciprocally and cannot be co-expressed within a T cell [43], [44]. However, it was recently reported that the expression of GATA-3 could be induced in Th1 cells expressing T-bet, and that several kinds of Th2-related cytokines, including IL-13, can be released from the Th1 cells [45]–[47]. This prompted us to investigate the expression of T-bet and GATA-3 in a hepatic γ4 or γ13 cells induced during the transition phase of *S. mansoni* infection. As exhibited in *Figure 8*, large proportions of both γ4 and γ13 cells expressed T-bet and GATA-3 simultaneously. This strongly suggests that the combined expression of T-bet and GATA-3 within a single γ4 or γ13 cell should play a definitive role in the simultaneous production of IFN-γ and IL-4 or IFN-γ and IL-13, respectively.

## Discussion

The generations of Th1 and Th2 immune responses are mutually repressed, and, in general, Th1- and Th2-related cytokines, especially IFN-γ and IL-4, the most typical Th1 and Th2 cytokines respectively, cannot be simultaneously secreted from one T cell [21], [22]. However, according to several previous reports [12], [13], [18]–[20], Th1- and Th2-immune conditions coexist in a *S. mansoni*-infected host, particularly in the liver when symptoms are not severe. We interrogated the previously unidentified cellular and/or molecular mechanisms allowing the coexistence of Th1 and Th2 in the liver during the transition phase of *S. mansoni* infection, the period between early Th1- and late Th2-dominant phases.

We show that *S. mansoni* infection induces the accumulation of unique CD4^+^ T cell populations in the liver of mice during the transition phase. These hepatic T cells produce uncommon combinations of cytokines, “IFN-γ and IL-13” and “IFN-γ and IL-4”. It was notable that T cells triply positive for IL-13, IL-4, and IFN-γ were also induced in the liver. Furthermore, these hepatic CD4^+^ T cells did not indiscriminately produce Th2-related cytokines, rather they preferentially produce a specific Th2 cytokines, as IL-5 was not produced.

Recently, it was reported that γδ T cells can acquire the potential to simultaneously produce IFN-γ and IL-4 [48]. As shown in *Figure 6*, both γ13 and γ4 cells that were induced during the transition phase of *S. mansoni* infection expressed αβ TCR, but not γδ TCR. DX5-negative T cells produced the combinations of “IFN-γ and IL-13” or “IFN-γ and IL-4” (Fig. 7). Not only DX5-negative T cells, but also DX5-positive T cells simultaneously produced these cytokines. As some NKT cells were reported to possess the ability to dually secrete IFN-γ and IL-4 [49], [50], NKT cells may be involved in such hepatic γ13 and γ4 cells. ICS with α-galactosylceramide/CD1d tetramer upon the hepatic CD4^+^ T cells or the usage of NKT cell-deficient mice, such as *cd1d^−/−^* or *jα18^−/−^* mice, may elucidate it. The expression of DX5 can be induced and up-regulated upon conventional DX5-negative T cells after activation [51], [52]. In addition, as shown in *Figure 1B*, most of the hepatic T cells were negative for CD62L and displayed an activated phenotype. Therefore, DX5-positive hepatic T cells, which showed unique cytokine profiles, would contain not only NKT cells but also conventional T cells upon which DX5 expression was induced. Collectively, conventional T cells are able to acquire the potential to produce uncommon combinations of cytokines during *S. mansoni* infection.

Although T-bet and GATA-3 should be important for the induction of the uncommon cytokine productivities upon the hepatic γ4 and γ13 cells (Figure 8), a possibility of the involvement of another transcription factor, promyelocytic leukemia zinc finger (PLZF), may not be excluded, as PLZF plays an important role in the exertion of the functions of γδ T cells and NKT cells that dually produced IFN-γ and IL-4 [48]–[50]. Furthermore, PLZF-transgenic T cells produced IFN-γ and IL-4 concomitantly upon TCR ligation [53]. As ectopic expression of PLZF seems to convert differentiated T cells into ‘innate’ type cells [53], [54], PLZF might have a function to reset the commitment to Th1 and Th2 cells.

T follicular helper cells (Tfh) are another T cell subset that can produce IFN-γ and IL-4 [55]–[58]. However, it remains controversial whether Tfh can produce IFN-γ and IL-4 “simultaneously”, and whether IFN-γ-producing Tfh cells and IL-4-producing Tfh cells are different sub-populations. Thus far, IL-13 production by Tfh has not been reported. Actually, the expression of the representative Tfh markers (CXCR5, ICOS, bcl-6) were rarely detected upon the hepatic γ4 and γ13 cells during the transition phase of *S. mansoni* infection (Fig. S8, data not shown). Moreover, no follicular structures were observed in the livers of *S. mansoni*-infected mice by histological analysis (data not shown). Therefore, it is unlikely that Tfh cells are the major population of the *S. mansoni-*induced hepatic γ4 and γ13 cells.

To our knowledge, this is the first report demonstrating conventional T cells simultaneously producing IFN-γ and IL-4, typical Th1 and Th2 cytokines, respectively. There are also very few other reports of T cells secreting triple cytokines IFN-γ, IL-13 and IL-4. Indeed, IL-18-elicited Th1 cells produced not only IFN-γ but also several Th2-related cytokines, including IL-13, and the Th2 cytokine-producing Th1 cells are termed as “super Th1 cells” [45], [46], [59]. Yet, super Th1 cells were reported not to secrete IL-4 [46]. Accordingly, the hepatic γ13 cells induced during *S. mansoni* infection may correspond to super Th1 cells, the γ4 cells and the triple positive cells were likely to be distinct from them.

Unfortunately, the roles of the unique CD4^+^ T cells described here upon the pathology of schistosomiasis are still unclear, as a method to specifically deplete or isolate these cells has not been established. *In vitro* culture system for the generation of T cells with the uncommon cytokine profiles is also unestablished. One promising way to establish such a method is through the identification of unique marker(s) for the population. Other ways are to understand the mechanisms of differentiation to, or expansion of, the unique hepatic T cell populations, which remain to be elucidated. The machinery of the induction and regulation of PLZF in T cells is unknown. One attractive way is using IL-18-deficient mice since IL-18 stimulation converts Th1 cells to super Th1 cells, that express GATA-3 and secrete IL-13, as mentioned above. However, super Th1 cells are not IL-4-producing cells. Therefore, it is conceivable that factors other than IL-18 are essential for the differentiation to the γ4 and the triple positive cells induced after *S. mansoni* infection, although some other cytokines and/or liver-specific microenvironments are likely to be required. Further studies are necessary for clarifying the role of these uncommon hepatic T cells in schistosomiasis.

It remains unclear the origin of this unique cell population; naïve T cells, antigen-specific Th1 cells, or Th2 cells. As mentioned above, PLZF-transgenic T cells that are not committed to Th1 or Th2 turn to be γ4 cells [53]. Therefore, conventional naïve T cells might possess inherently the potential to acquire the unique cytokine profiles although it has not been reported whether PLZF is induced in conventional T cells under biological condition. Meanwhile, the uncommon T cells reported here proliferate in the liver, but not in the spleen, during transition phase of the infection, when oviposition begins (4–5 weeks PI, data not shown). This may indicate the importance of the antigens derived from the maturing worm and/or the egg. At present, it is ambiguous whether the unique T cells described here expanded mono-, oligo-, or poly-clonally. Recently, omega-1, a glycoprotein which is secreted from *S. mansoni* eggs and which is most abundantly present in soluble egg antigen, has been demonstrated to condition dendritic cells (DCs) to prime Th2 responses [60]–[62]. In these reports, naïve T cells were co-cultured with omega-1-immunized DCs and differentiation to Th2 cells was demonstrated. Since the unique hepatic T cells increased during the transition phase of *S. mansoni* infection, it would be worthwhile to analyze the fate of Th1 cells, not Th2 cells, stimulated with omega-1-immunized DCs, but we cannot exclude the possibility that Th2 cells changed their nature. In addition, the effect of omega-1 upon antigen-presenting cells in a liver, such as hepatic DCs, Kupffer cells, and hepatocytes [63], also warrants investigation.

In summary, these data shed light on the etiology of schistosomiasis. Furthermore, these results also put forward a novel concept in T cell biology; that the commitment to Th1 or Th2 might be reset in some immunological conditions.

## Supporting Information

Figure S1 **Varieties of immune competent cells were found in the liver after** ***S. mansoni*** **infection.** (A-C) Hepatic cells were isolated at indicated time points from 3 BALB/c mice and were pooled for conducting following flowcytometric analysis. (A) The percentages represent the proportions of B220^+^ CD19^+^ cell population. (B) The percentages indicate the proportions of DX5^+^ cells in CD3-negative population. (C) The percentages show the proportions of Siglec-F^+^ population in the cells with CD45^+^ CD11c^low/−^ staining profile. (A-C) Similar results were obtained in three independent experiments.
(TIF)

Figure S2 ***S. mansoni*** **infection elicited various cytokine production, except for IL-17 production, upon hepatic T cells.** Hepatic lymphocytes were isolated from *S. mansoni*-infected mice at indicated time points, and their potential for producing TNF-α, IL-10, IL-5, or IL-17A was analyzed by ICS upon TCR ligation. Insets at the top represent one example using liver lymphocytes prepared at 4 weeks PI. The values represent the percentages in CD4-positive population. This experiment is representative of three independent experiments.
(TIF)

Figure S3 ***S. mansoni*** **infection induced production of IFN-γ but neither IL-4 nor IL-13 from hepatic CD8^+^ T cells.** Hepatic lymphocytes were isolated from *S. mansoni*-infected mice at 6 weeks PI, and ICS was conducted for investigating the potential of CD8^+^ T cells to produce IFN-γ, IL-4, or IL-13. One example representative result is shown. The percentages in the insets represent the proportions in CD8-positive population. Similar results were obtained in five independent experiments.
(TIF)

Figure S4 **Neither γ13 nor γ4 cells were induced in the spleens of** ***S. mansoni*** **-infected mice.** (A and B) Hepatic lymphocytes and splenocytes were isolated from *S. mansoni*-infected mice at indicated time points, and ICS was conducted after TCR stimulation. (A) One example using hepatic lymphocytes prepared at 6 weeks PI is displayed. The percentages in the insets represent the proportions in CD4-positive population. (B) The proportions of γ4 cells (upper graph) or γ13 cells (lower graph) in CD4-positive hepatic or splenic lymphocytes were investigated. Similar results were obtained in three independent experiments.
(TIF)

Figure S5 **The unique T cells were induced in the livers of** ***S. mansoni*** **-infected C57BL/6 mice.** (A and B) Hepatic lymphocytes were isolated from *S. mansoni*-infected BALB/c or C57BL/6 mice at indicated time points, and ICS was conducted upon TCR ligation. (A) One example using liver lymphocytes prepared at 6 weeks PI is exhibited. (B) The proportions of γ4 cells (upper graph) or γ13 cells (lower graph) in CD4-positive hepatic lymphocytes were investigated. This experiment is representative of three independent experiments.
(TIF)

Figure S6 **TCR ligation is required for the induction of cytokine production by the hepatic lymphocytes.** In the absence of TCR stimulation, ICS was conducted using fresh hepatic lymphocytes isolated from *S. mansoni*-infected mice at 6 weeks PI. This experiment is representative of two independent experiments.
(TIF)

Figure S7 **The hepatic γ4 and γ13 cells showed effector memory T cell-like surface phenotypes.** Hepatic lymphocytes were isolated from *S. mansoni*-infected mice at 6 weeks PI, and ICS was conducted after TCR stimulation. The percentages in the insets represent the proportions in γ4 or γ13 cells. Similar results were obtained in two independent experiments.
(TIF)

Figure S8 **Both γ4 and γ13 cells little expressed the surface markers of Tfh cells.** Hepatic lymphocytes were isolated from *S. mansoni*-infected mice at 6 weeks PI and ICS was conducted upon TCR ligation. The percentages in the insets represent the proportions in γ4 or γ13 cells. One representative result of two independent experiments is shown.
(TIF)
