# Supplementary figures and images for: Unique T Cells with Unconventional Cytokine Profiles Induced in the Livers of Mice during Schistosoma mansoni Infection
Source: PLoS One. 2013 Dec 16;8(12):e82698. doi: 10.1371/journal.pone.0082698 (PMC3865148; doi:10.1371/journal.pone.0082698)

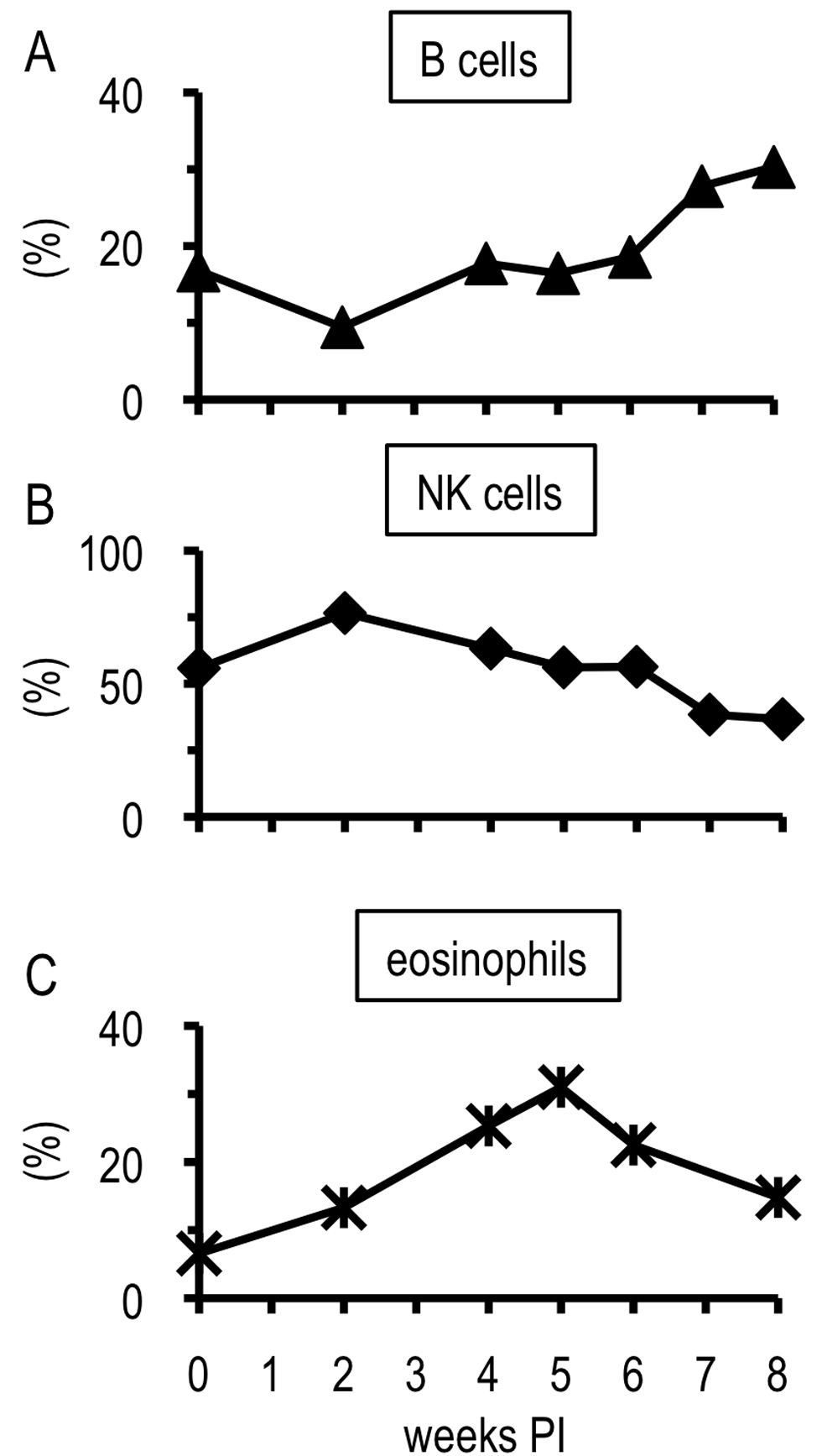

Supplement: Figure S1 — Varieties of immune competent cells were found in the liver after S. mansoni infection. (A-C) Hepatic cells were isolated at indicated time points from 3 BALB/c mice and were pooled for conducting following flowcytometric analysis. (A) The percentages represent the proportions of B220+ CD19+ cell population. (B) The percentages indicate the proportions of DX5+ cells in CD3-negative population. (C) The percentages show the proportions of Siglec-F+ population in the cells with CD45+ CD11clow/− staining profile. (A-C) Similar results were obtained in three independent experiments. (TIF) [file pone.0082698.s001.tif]

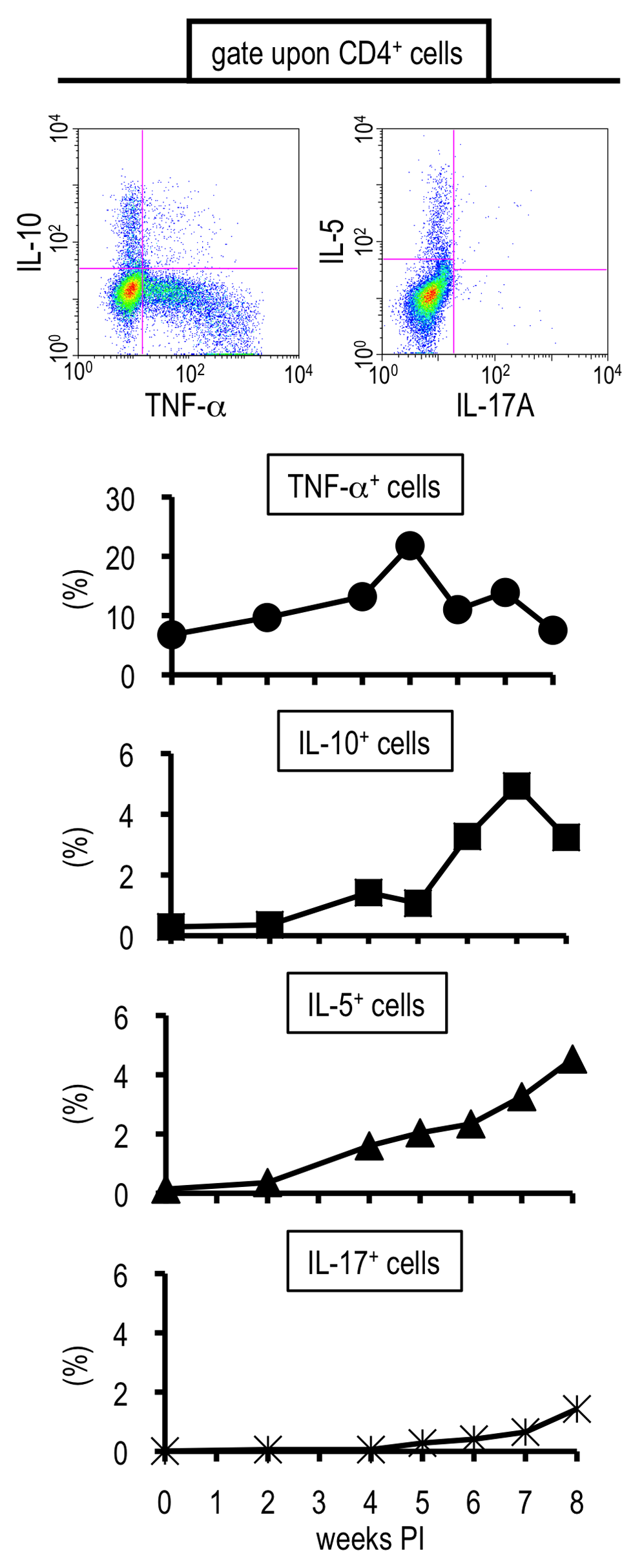

Supplement: Figure S2 — S. mansoni infection elicited various cytokine production, except for IL-17 production, upon hepatic T cells. Hepatic lymphocytes were isolated from S. mansoni-infected mice at indicated time points, and their potential for producing TNF-α, IL-10, IL-5, or IL-17A was analyzed by ICS upon TCR ligation. Insets at the top represent one example using liver lymphocytes prepared at 4 weeks PI. The values represent the percentages in CD4-positive population. This experiment is representative of three independent experiments. (TIF) [file pone.0082698.s002.tif]

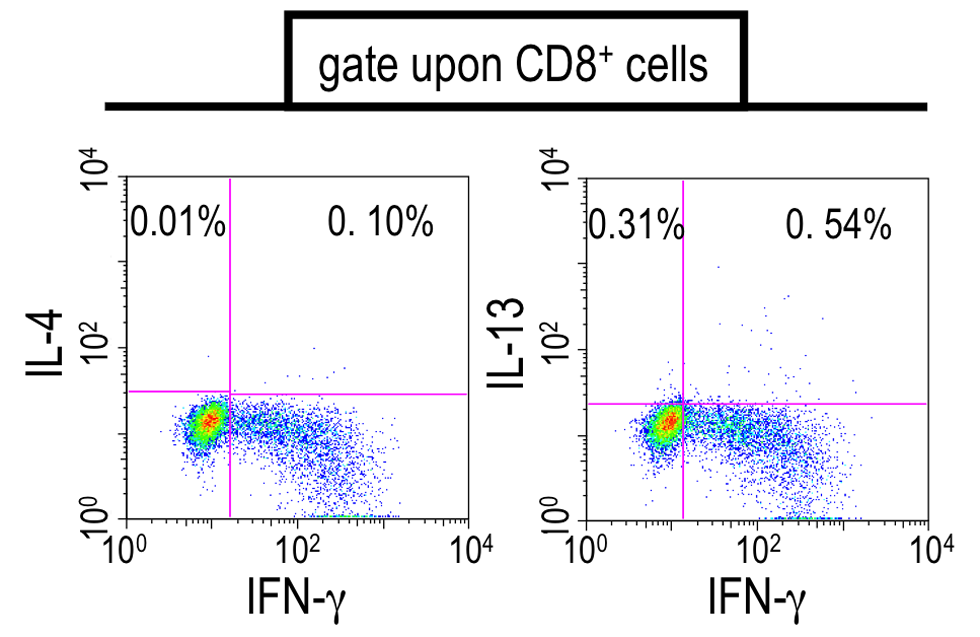

Supplement: Figure S3 — S. mansoni infection induced production of IFN-γ but neither IL-4 nor IL-13 from hepatic CD8+ T cells. Hepatic lymphocytes were isolated from S. mansoni-infected mice at 6 weeks PI, and ICS was conducted for investigating the potential of CD8+ T cells to produce IFN-γ, IL-4, or IL-13. One example representative result is shown. The percentages in the insets represent the proportions in CD8-positive population. Similar results were obtained in five independent experiments. (TIF) [file pone.0082698.s003.tif]

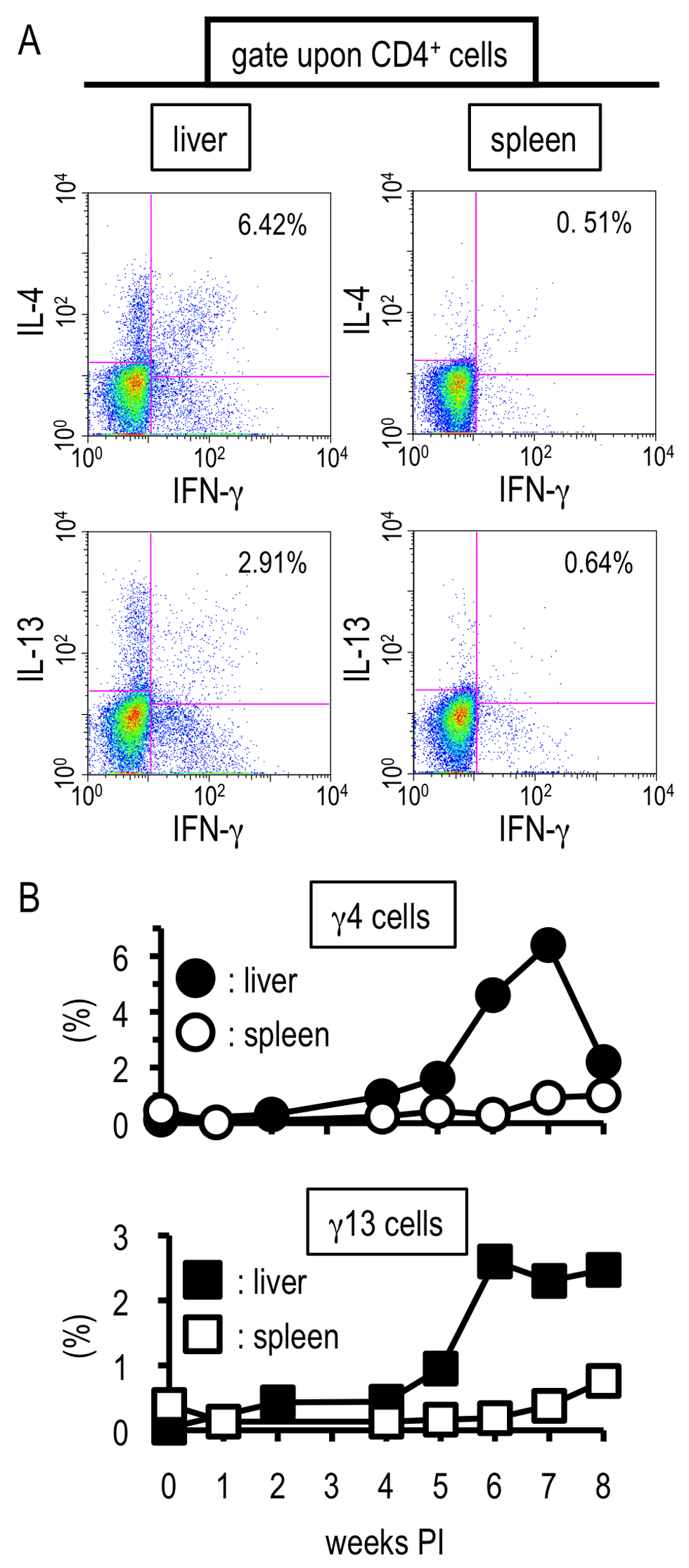

Supplement: Figure S4 — Neither γ13 nor γ4 cells were induced in the spleens of S. mansoni -infected mice. (A and B) Hepatic lymphocytes and splenocytes were isolated from S. mansoni-infected mice at indicated time points, and ICS was conducted after TCR stimulation. (A) One example using hepatic lymphocytes prepared at 6 weeks PI is displayed. The percentages in the insets represent the proportions in CD4-positive population. (B) The proportions of γ4 cells (upper graph) or γ13 cells (lower graph) in CD4-positive hepatic or splenic lymphocytes were investigated. Similar results were obtained in three independent experiments. (TIF) [file pone.0082698.s004.tif]

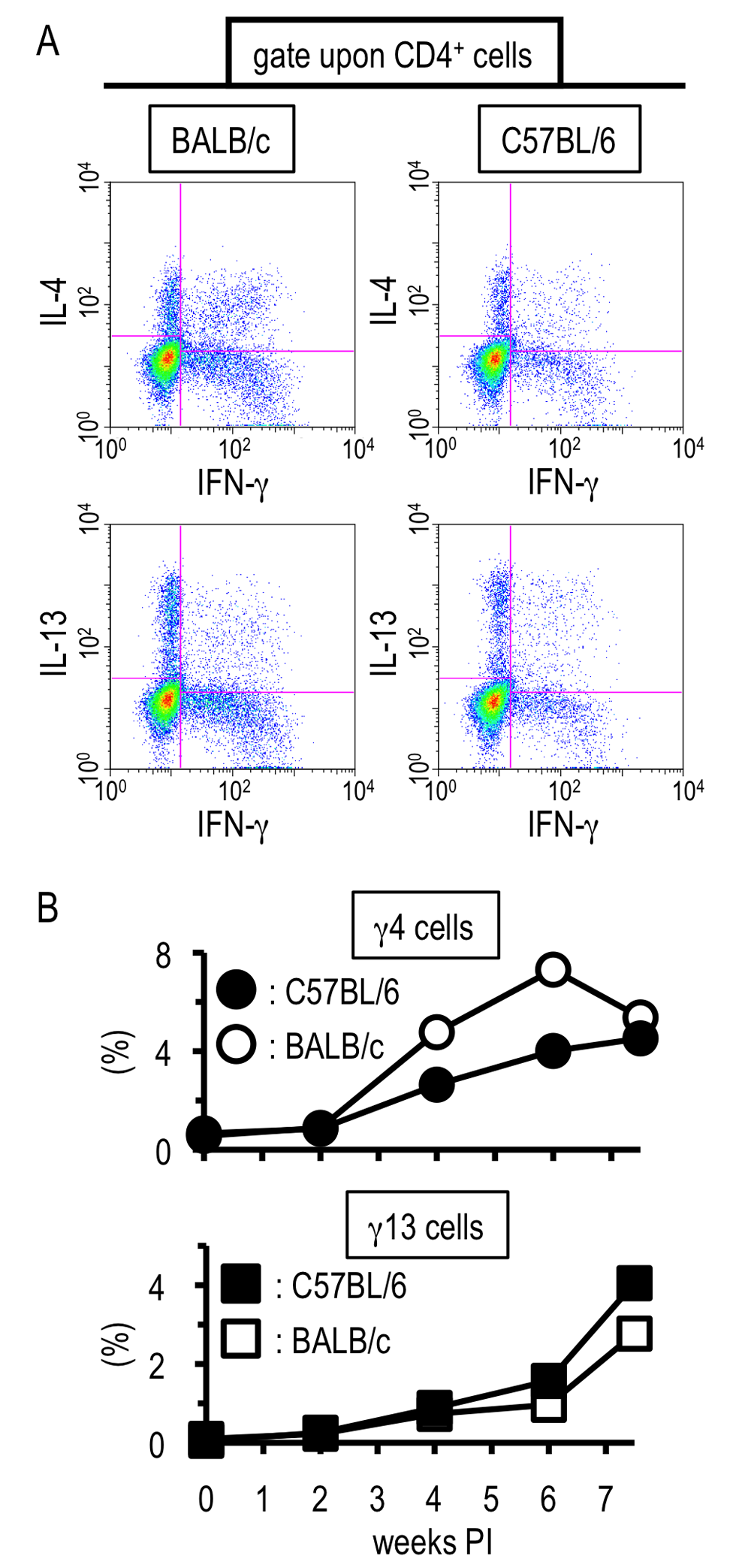

Supplement: Figure S5 — The unique T cells were induced in the livers of S. mansoni -infected C57BL/6 mice. (A and B) Hepatic lymphocytes were isolated from S. mansoni-infected BALB/c or C57BL/6 mice at indicated time points, and ICS was conducted upon TCR ligation. (A) One example using liver lymphocytes prepared at 6 weeks PI is exhibited. (B) The proportions of γ4 cells (upper graph) or γ13 cells (lower graph) in CD4-positive hepatic lymphocytes were investigated. This experiment is representative of three independent experiments. (TIF) [file pone.0082698.s005.tif]

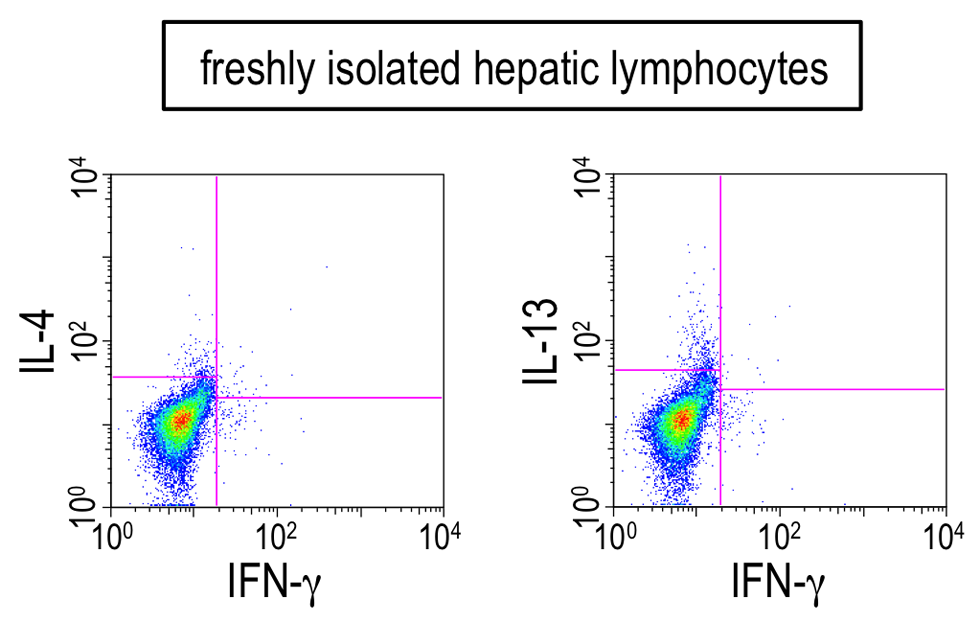

Supplement: Figure S6 — TCR ligation is required for the induction of cytokine production by the hepatic lymphocytes. In the absence of TCR stimulation, ICS was conducted using fresh hepatic lymphocytes isolated from S. mansoni-infected mice at 6 weeks PI. This experiment is representative of two independent experiments. (TIF) [file pone.0082698.s006.tif]

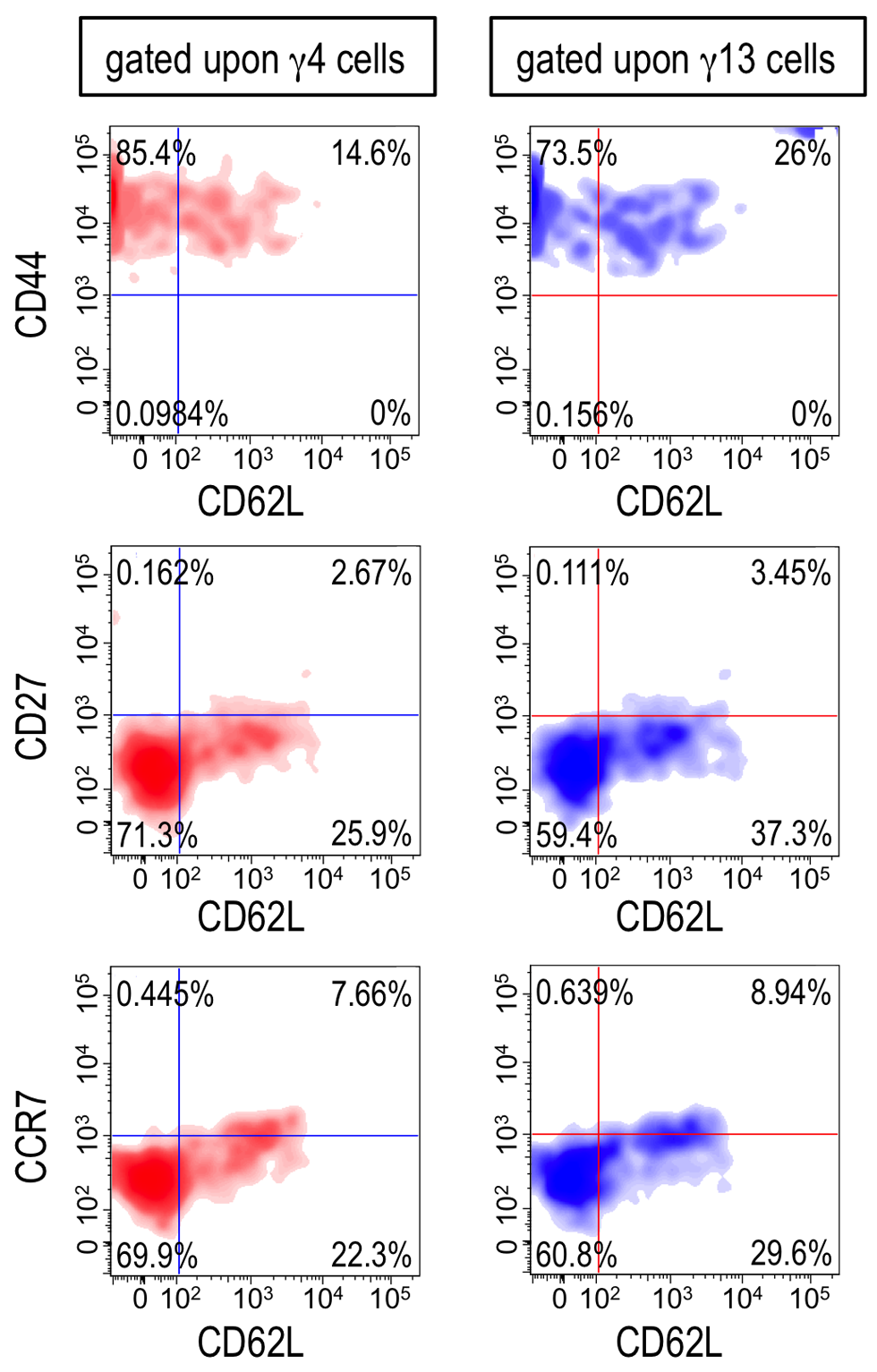

Supplement: Figure S7 — The hepatic γ4 and γ13 cells showed effector memory T cell-like surface phenotypes. Hepatic lymphocytes were isolated from S. mansoni-infected mice at 6 weeks PI, and ICS was conducted after TCR stimulation. The percentages in the insets represent the proportions in γ4 or γ13 cells. Similar results were obtained in two independent experiments. (TIF) [file pone.0082698.s007.tif]

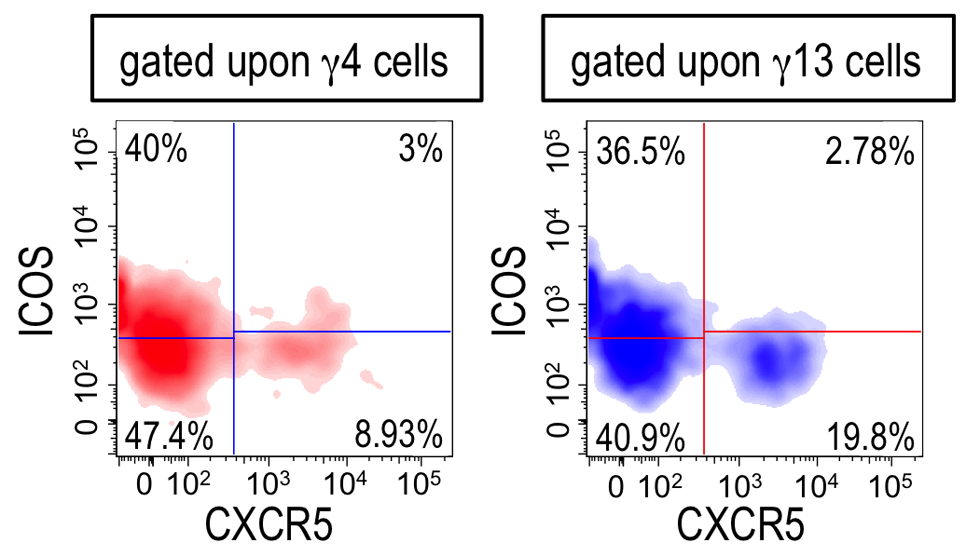

Supplement: Figure S8 — Both γ4 and γ13 cells little expressed the surface markers of Tfh cells. Hepatic lymphocytes were isolated from S. mansoni-infected mice at 6 weeks PI and ICS was conducted upon TCR ligation. The percentages in the insets represent the proportions in γ4 or γ13 cells. One representative result of two independent experiments is shown. (TIF) [file pone.0082698.s008.tif]
